# Spike-Based Reinforcement Learning in Continuous State and Action Space: When Policy Gradient Methods Fail

**DOI:** 10.1371/journal.pcbi.1000586

**Published:** 2009-12-04

**Authors:** Eleni Vasilaki, Nicolas Frémaux, Robert Urbanczik, Walter Senn, Wulfram Gerstner

**Affiliations:** 1 Laboratory of Computational Neuroscience, EPFL, Lausanne, Switzerland; 2 Department of Computer Science, University of Sheffield, Sheffield, United Kingdom; 3 Department of Physiology, University of Bern, Bern, Switzerland; University College London, United Kingdom

## Abstract

Changes of synaptic connections between neurons are thought to be the physiological basis of learning. These changes can be gated by neuromodulators that encode the presence of reward. We study a family of reward-modulated synaptic learning rules for spiking neurons on a learning task in continuous space inspired by the Morris Water maze. The synaptic update rule modifies the release probability of synaptic transmission and depends on the timing of presynaptic spike arrival, postsynaptic action potentials, as well as the membrane potential of the postsynaptic neuron. The family of learning rules includes an optimal rule derived from policy gradient methods as well as reward modulated Hebbian learning. The synaptic update rule is implemented in a population of spiking neurons using a network architecture that combines feedforward input with lateral connections. Actions are represented by a population of hypothetical action cells with strong mexican-hat connectivity and are read out at theta frequency. We show that in this architecture, a standard policy gradient rule fails to solve the Morris watermaze task, whereas a variant with a Hebbian bias can learn the task within 20 trials, consistent with experiments. This result does not depend on implementation details such as the size of the neuronal populations. Our theoretical approach shows how learning new behaviors can be linked to reward-modulated plasticity at the level of single synapses and makes predictions about the voltage and spike-timing dependence of synaptic plasticity and the influence of neuromodulators such as dopamine. It is an important step towards connecting formal theories of reinforcement learning with neuronal and synaptic properties.

## Author Summary

Humans and animals learn if they receive reward. Such reward is likely to be communicated throughout the brain by neuromodulatory signals. In this paper we present a network of model neurons, which communicate by short electrical pulses (spikes). Learning is achieved by modifying the input connections depending on the signals they emit and receive, if a sequence of action is followed by reward. With such a learning rule, a simulated animal learns to find (starting from arbitrary initial conditions) a target location where reward has occurred in the past.

## Introduction

Animals can learn new behaviors by exploring available actions in the presence of reward signals. Typical conditioning experiments are structured so that animals learn by trial and error, either by reinforcing a desired behavior with a positive reward (finding food, escaping from a stressful situation), or by penalizing undesired actions by a negative reward signal (electric shock or uncomfortable water temperature). Learning by reward is known in the field of machine learning as reinforcement learning [1] but has roots in behavioral psychology that can be traced back at least to Thorndike's law of effect [2]. These early ideas have influenced the mathematical description of classical conditioning in the theories of Rescorla and Wagner [3], the ‘hedonistic neuron’ of Klopf [4],[5], or the early psychological theories of animal learning and conditioning by Sutton and Barto [6]–[8]. Before we turn to the specific learning paradigm that we consider in the present paper, we devote some space in this introduction section to an extensive review of three-factor rules in spiking neuron models and their relation to unsupervised Hebbian models and classical reinforcement learning models. The contributions of the present paper are sketched on the background of this earlier work.

### 

#### Didactic Review of three-factor rules

On the cellular level, learning and memory is thought to be implemented by changes in the strength of the synaptic connection between pairs of neurons [9],[10]. Many of the classical experiments on Long-Term Potentiation and Depression (LTP and LTD) have been inspired by the ideas of Hebb that the co-activation of two neurons should lead to a strengthening of the connection between them [11]. Thus, according the Hebb's principle the change of a weight 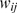 from a presynaptic neuron 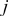 to a postsynaptic neuron 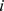 depends only on the state of the presynaptic and postsynaptic neurons
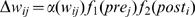
(1)with some learning rate 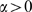. Even without specifying the functions 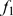 and 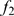 and the exact nature of the states 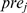 and 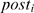 of the two neurons, the equation (1) captures the essence of a Hebb rule, i.e., the weight change depends only on the state of the two neurons, and possibly on the current value of the weight itself, but not on that of other neurons or other signals. Such a ‘2-factor’ Hebb rule is the basis of classical models of unsupervised [12],[13] and developmental learning [14],[15]. In these classical models the functions 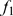 and 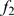 are linear or quadratic functions of the firing rates of pre- and postsynaptic neurons, respectively. Modern models of Spike-Timing Dependent Plasticity (STDP) can be considered as an implementation of Hebb's rule on the level of spikes [16]–[21].

However, a Hebbian two-factor rule, be it formulated on the level of spikes or on the level of rates, cannot take into account the presence or absence of a reward signal. Rewarding situations are thought to be represented in the brain by changes in the concentration of neuromodulators that is available to and shared by large populations of neurons. More precisely, in some brain areas, dopamine has been identified as candidate molecule signaling unexpected rewarding situation [22]. It is therefore tempting to extend the ‘local’ Hebbian rule in Eq. (1) by a third factor 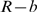, where 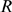 represents a ‘global’ neuromodulatory signal characterizing rewarding situations and 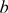 a baseline
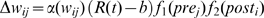
(2)Suppose for the moment that 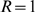 if the animal has recently received a reward and 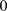 otherwise and 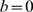. The consequence of the 3-factor rule (2) is that a weight change predicted by the Hebbian rule (1) is implemented only in the presence of a reward. In the absence of reward, a weight change cannot occur.

Experimentally, three-factor rules such as (2) have been studied extensively in the cortico-striatal synapse [23]–[26] using a classical firing rate-based protocol. A different line of research around synaptic tagging [27] in the hippocampus has shown that synaptic changes induced by tetanic protocols of Long-Term-Potentiation can be stabilized only in the presence of neuromodulators such as dopamine [28]–[30] suggesting that the Hebbian changes need neuromodulators as a third factor for stabilization. More recently the timing-dependence of the three factor rule in cortical-striatal synapses has been studied on the level of spikes, yielding a form of dopamine-dependent STDP [31].

Theories of three-factor rules on the time scale of milliseconds have been addressed by a number of different groups [32]–[37]. Three different theoretical approaches can be distinguished. The first one consists in deriving a learning rule from reward optimization by gradient descent [32]–[34],[38], an approach that can be linked to policy gradient methods in machine learning [39],[40]; the second one postulates a form of STDP that is modulated by reward [33],[35],[36],[41], an approach that can be considered an extension of classical STDP models [16],[17],[42]; the third one translates the framework of Temporal-Difference learning (TD) models [1],[43], in particular actor-critic models [1],[7],[44], to spiking neuronal networks [37],[45]. As an aside, gradient rules can be also formulated in the context of node and weight perturbation where the postsynaptic activity does not explicitly enter, yielding a modified two-factor rule rather than a three-factor rule [46],[47]. We would also like to mention the sensitivity of STDP to the derivative of the postsynaptic activity which has been related to TD-learning [48]–[50].

In this paper we study a network of spiking neurons that has to solve a navigation problem to a hidden target. Rewards are delayed, i.e., the animal has to perform a sequence of action before it receives a positive or negative reward signal. Our approach can be related to policy gradient methods for spiking neurons [32]–[34], but goes beyond these earlier studies for two reasons: First, we consider a more general class of learning rules that contain policy gradient rules and a naive reward modulated Hebbian rule as a special case. Second, we consider the case of strong lateral interaction between action neurons, that lead to the spontaneous formation of activity bumps in the layer where the action selection takes place.

The resulting synaptic update rules can be formulated as a differential equation in continuous time that has the form of a three-factor rule
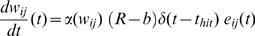
(3)

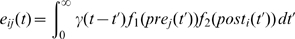
(4)The term 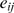, called eligibility trace, picks up the correlations between pre- and postsynaptic activity just as in a Hebbian learning rule and convolves these with a low-pass filter 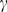. However, the final weight change is implemented only in the presence of a reward signal 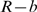 which is delivered at the time 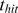 when the animal hits the target. The choices of 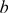 considered in this paper are: 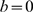 and 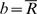, where 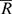 is the reward signal averaged over many trials.

In contrast to earlier work of Xie and Seung [32] but similar to [33]–[35] our approach takes into account spiking neurons with refractoriness and includes examples such as the standard integrate-and-fire model. Under certain conditions on the refractoriness [34], our learning rule can be identified with a standard STDP model, but modulated by a third factor [33]–[36]. In contrast to most earlier work [33],[34],[36], our learning rule is applied to a network of neurons that combines feed-forward input with lateral interactions.

#### Learning paradigm

In order to show the potential of the family of spike-timing dependent three-factor rules studied in this paper, we apply it to the Morris water maze paradigm [51]. It is a standard paradigm of behavioral learning and navigation, and has also already been used as a challenging paradigm for TD-learning models [52]–[55]. In this behavioral paradigm, a rat (or mouse) is placed in a pool of milky (non-transparent) water. In order to escape from the water, it has to find an invisible platform hidden just below the water surface. Climbing on the hidden platform can be considered as rewarding, since it ends a disagreeable experience. During the first trial of the experiment, the rat discovers the platform by chance. In subsequent trials the rat is each time placed at a different starting location. Nevertheless, across several trials the rat learns to navigate towards the hidden platform based on distal surrounding cues [52],[56]. In contrast to a variant of the task with *fixed* initial condition [57], the Morris Watermaze task with *variable* starting condition considered in this paper depends on the hippocampus [51].

In this paper we model the Morris Watermaze paradigm using a minimal hippocampal model of spiking neurons. The model we propose has the following features:

1. The position of the rat is a continuous quantity represented by an ensemble of place cells with overlapping place fields (coarse coding). These place cells have feedforward connections to action cells.
2. Actions are represented by a population of action cells representing different direction of movements in a coarse coding paradigm. New actions, defined as the population vector activity across action cells, are chosen periodically at theta frequency.
3. The action cells are organized on a ring with lateral connectivity showing local excitation and long-range inhibition. As a result, the population of action cells respond to input from place cells with a bump-like activity profile.
4. The feedforward connections of place cells to action cells change according to a three factor learning rule on the level of spikes, that can be considered reward modulated form of Hebbian plasticity derived from reward maximization.
5. Synaptic transmission in the feedforward connections is stochastic and learning takes place through the modification of the release probability.
6. The problem of learning a sequence of actions when reward is given at only the end of the sequence is solved by an eligibility trace that appears naturally in the derivation of the learning rule. The eligibility trace is implemented as a local memory at the site of the synapse.

A large fraction of classical reinforcement models have been developed for artificial systems with a finite number of (discrete) states and a small number of actions. However, real animals move in a continuous space and, in some paradigms, also have a large choice of actions that is best described as a continuum. Classical TD models such as Q-learning [1],[43], are ill adapted to this situation: if a continuous state is approximated by a discretized state-space of increasing resolution (larger number of states) learning slows down, unless an eligibility trace is introduced into the algorithm and/or function approximation is used [1]. On the contrary, the architecture we adopt here allows the animal to move in a continuous arena, without a significant reduction in performance.

Moreover, while convergence of TD models is guaranteed in the presence of an eligibility trace [58],[59], the addition of an eligibility trace in these algorithm is somewhat *ad hoc*, whereas eligibility traces appear naturally in the policy gradient framework. Surprisingly, the standard policy gradient method for spiking neurons [32],[34] does not work for the scenario where action choices are decided by the formation of an activity bump in the layer of action cells. However, we will show that our model network with a modified learning rule with a ‘Hebbian bias’ does learn navigation to an invisible goal within 20 trials, similar to the performance of rats in the Morris Water Maze task [52]. Because of the coarse coding of states and actions by cells with overlapping place fields and ‘action fields’, the model allows to encode position and action in continuous state and action spaces. We will show that with our coarse coding approach the learning performance is independent of the number of cells. Thus performance is stable and does not depend on implementation details. We argue that on one hand, a crucial ingredient of this structural stability are the lateral interactions in the ring of action cells; on the other hand it is exactly the fact that actions are chosen based on the location of a stable activity bump that makes standard policy gradient methods fail.

## Results

The results section is organized in three main parts. First, we discuss the main features of our three-factor learning rule for spiking neurons. To test this learning rule in a realistic paradigm, we introduce in the second part the Morris water-maze learning task and the model architecture with place cells and action cells suitable for solving the task. Finally, the performance of the learning rule in this task is presented.

### Three-factor learning rule for spiking neurons

We consider a Spike Response Model neuron with index 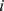 that receives input from other neurons 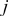. The 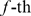 input spike from neuron 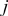 arrives at time 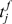 at a synapses onto neuron 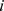 and causes there an excitatory (or inhibitory) postsynaptic potential (EPSP or IPSP) of time course 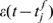 and amplitude 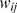. The EPSPs and IPSPs of all incoming spikes are added to the membrane potential 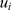 of neuron 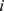. Spikes are generated stochastically with an instantaneous rate (or stochastic intensity)
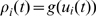
(5)where 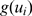 is a positive function that increases with the membrane potential 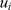, see also Eq. (24). Immediately after a spike of neuron 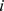 at time 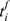, the neuron enters into a state of relative refractoriness, which is implemented by a hyperpolarizing spike afterpotential 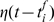. Thus the total membrane potential of the Spike Response Model neuron is [20]:
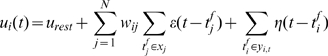
(6)where 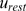 is the resting potential, 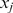 is the set of presynaptic spikes, 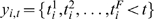 is the set of postsynaptic spikes up to time 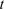.

Using this neuron model, we can calculate the probability that neuron 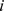 generates a specific spike train with firing times 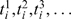 during a trial of duration 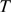 [34], see Methods, Eq. (25). Some of the spikes of neurons 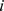 occur just before a reward is delivered, others not. The aim of learning is to change the synaptic weights 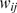 so that the probability of receiving a reward 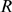 increases. We consider learning rules of the form
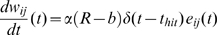
(7)where 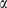 is the learning rate (controlling the amplitude of weight updates), 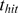 the moment when the animal hits the target or the wall, 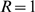 is the positive reward for finding the target, 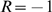 the (negative) reward for bumping into a wall and b a reward baseline, for instance an estimate of the positive reward based on past experience. The eligibility trace 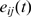 evolves according
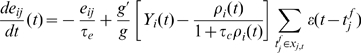
(8)where 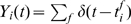 is the spike train of the postsynaptic neuron, 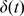 the Dirac function, 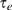 the eligibility trace time constant, 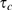 a parameter with units of time, and 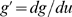 the derivative of the function 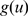.

Because of the parameter 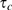, the learning equations (9) and (8) define a *family* of learning rules, rather than one single instance of a rule. The parameter 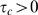 is a specific feature of our model which allows to turn the model from a strict policy gradient method (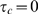, [33],[34] see methods) to a naive Hebbian model (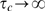, see below the discussion of the postsynaptic factor). Thus we are able to link and compare these conceptually different rules via the modification of 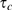. We note that for small firing rates 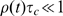, Eq. (9) approximates the optimal policy gradient rule of [33],[34], while for larger firing rates, it enhances the Hebbian component of the rule. For 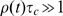, the term in the square brackets goes to 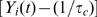 so that for 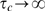 learning is driven by the Hebbian correlation term 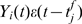. In the main body of the simulation results, we pick a fixed value of 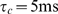 which implies that we use a policy gradient method with a Hebbian bias.

The estimate of the positive reward is calculated as a running mean updated *at the end of the trial* according the following equation: 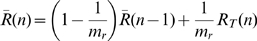, with 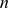 being the number of the trial and 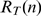 being the reward at the end of the 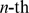 trial (1 or 0) and 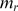 the width of the averaging window.

We will now show that Eqs. (7) and (8) can be interpreted as a three-factor learning rule for spiking neurons, within the general framework outlined in the introduction.

#### Presynaptic factor

Presynaptic spike arrival causes an EPSP. The time course of the EPSP 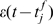 represents the effect of presynaptic activity at the location of the synapse. We emphasize that the term presynaptic factor does not imply that this factor is implemented presynaptically - rather it refers to a term causes by the activity of the presynaptic neuron 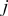.

#### Postsynaptic factor

**Figure 1:**
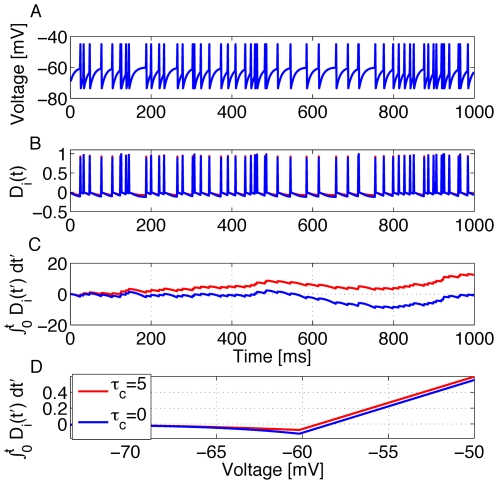
Postsynaptic factors of the learning rule. A model neuron receives constant strong input making it fire at about 50Hz. A:Time course of the voltage. B: The postsynaptic factor 
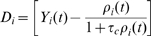
 of the rule evaluated in time steps of 1 ms (see Eq. 8). The postsynaptic factor decreases with voltage, but has a sharp positive peak during a spike. The case 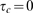 (blue line) and 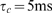 are nearly indistinguishable. C: The accumulated term 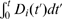 as a function of time 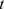 shows a clear difference between the two cases. For the model with 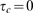 (blue line) it fluctuates around 0 while for the model with 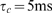 (red line) it exhibits a positive drift. D: The postsynaptic factor as a function of voltage is extracted from the data in graphs A and B by plotting the momentary value of 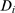 from graph B as a function of the voltage in graph A in the same time step. For voltages above 60 mV the neuron models always spikes for this input scenario, so that the postsynaptic factor is positive.

Postsynaptic activity is represented by both the timing 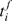 of postsynaptic action potentials and the postsynaptic membrane potential 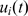. The membrane potential enters in the function 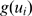 that determines the instantaneous firing rate 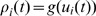. Postsynaptic spikes are treated as events and described by the function 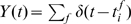. The postsynaptic factor, denoted by 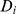, is encapsulated by the square brackets in Eq. (8) and visualized as a function of membrane potential in Figure 1. For the case of 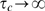 the postsynaptic factor depends only on spike timing, but not on the membrane potential of the postsynaptic neuron.

The presynaptic and postsynaptic factors both enter into the eligibility trace 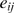 of Eq. (8) which is a quantity that must be stored locally at the synapses from neuron 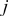 to neuron 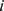. The eligibility trace of the synapse from 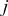 to 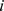 is updated by a finite positive amount whenever a postsynaptic action potential occurs within the time span of an EPSP at this synapse. Hence the eligibility trace picks up (potentially causal) correlations between presynaptic spike arrival and postsynaptic spike firing. If an EPSP occurs without a postsynaptic spike, the eligibility trace decays smoothly at a rate proportional to 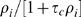. In particular, if the membrane potential is high, but no postsynaptic spike is triggered, the eligibility trace decreases strongly. However, in the limit 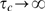 such a depression of the synapse does not occur. Thus, for 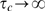 the eligibility trace is naive Hebbian in the sense that it is increased if postsynpatic spikes occur shortly after (and potentially triggered by) presynaptic spike arrival. If a synapse is not active (that is, in the absence of an EPSP at the synapse), the eligibility always decays with a slow time constant 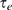 in the range of seconds. Whatever the choice of 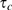, the eligibility trace uses only local quantities that are available at the site of the synapse and stores locally the correlations between pre- and postsynaptic activity averaged over several seconds. In the limit of 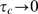 these correlations are zero *on average* because spikes 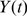 are generated at the rate 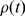 so that the expectation 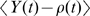 vanishes. However, in a single trial the correlations stored by the eligibility trace are typically nonzero.

#### Global factor

The third factor in our synaptic learning rule is the global reward term described by the expression 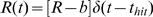. It represents in our theory the time course of the (external) reward delivery. Neuromodulators such as dopamine represent a diffusive reward-related signal across large brain regions [22]. In our theory, the synapse calculates and stores locally the eligibility trace. However, changes at the weights are implemented only, if the change ‘proposed’ by the eligibility trace is ‘confirmed’ by a global neuromodulatory signal.

#### Stochastic binary synapses

Transmission of information across the synapse is not a deterministic event, but has a stochastic component. Changes in the synaptic ‘weight’ 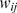 discussed above, are likely to correspond to changes in the probability 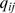 of releasing a fixed amount of neurotransmitter across the synaptic cleft [60]. Let us suppose that the synapse transmits either a fixed amount 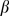 of neurotransmitter or nothing at all. Learning affects the neurotransmitter release so that increasing the weight 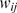 of the synapse by the above update rule will increase the release probability such that the mean weight can be expressed as 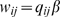. Thus, for stochastic binary synapses, as used in our simulations, we arrive at the following learning rule
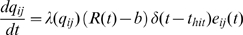
(9)where the eligibility trace is the same as in Eq. (8). Since 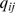 is a probability it is bounded to a maximum of 1. We also impose a lower bound 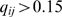. We implement these contraints by a learning rate 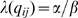 for 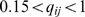 and zero otherwise.

#### Learning rule parameters

Free parameters are: the learning rate 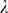, the eligibility trace time constant 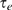, parameter 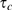, which tunes the Hebbian bias of the learning rule, and the noise level of the neuronal response (controlled by parameter 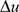, see Model architecture, Action Cells). Other parameters are fixed a priory [34],[61].

### Model architecture

**Figure 2:**
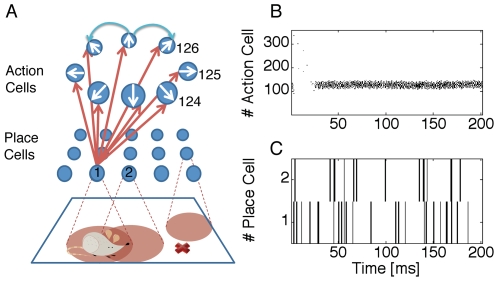
Hippocampal model. A: Schematic overview. Place cells are connected via all-to-all feedforward connections (red) to the action cells, which in addition receive lateral input (light blue) via connections with a mexican hat profile (not all connections shown). B: Rasterplot of action cells, showing activity of the cells encoding for the chosen direction. The spiking activity of action cells starts with stochastic firing at low rates until an activity bump is formed after 25ms. C: Spike train of neurons labeled 1 and 2, corresponding to the schema on the left, when the rodent is placed in the receptive field of neuron 1.

The learning rules discussed in the previous subsection (with 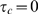, 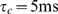 and 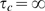) are tested on a simulated Morris Watermaze task with variable start condition, a task known to involve hippocampus [51]. Hippocampus is represented as a population of place cells, with place cells centers organized on a rectangular grid. These model place cells project onto ‘action’ cells, putatively placed in the nucleus accumbens. The population of action cells represents the next action to be chosen by the model rat and is organized in a ring-like topology with lateral connectivity of the Mexican-hat type; see Figure 2.

#### Hippocampal place cells (HPC)

Hippocampal place cells are modeled as Poisson neurons with a firing rate 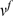 that is a Gaussian function of the animal position in the environment:
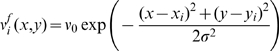
(10)where 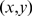 is the current position of the animal, 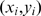 is the position at which the 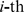 place cell gives the strongest response, 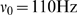 is the maximum firing rate of the place cell. Unless marked otherwise, we consider in our simulations 100 such neurons placed on a grid of 10×10 cells, with a distance of 10cm between two neighboring cells and with 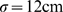 being the width of each place field. The environment is a box of 100×100 cm. The ensemble activity of place cells encodes the position 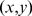 of the animal.

#### Action cells (AC)

Action cells are modeled as Leaky Integrate and Fire units [62], which are a special case of the Spike Response Model [20]. The change of the membrane potential of neuron 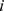 is given by
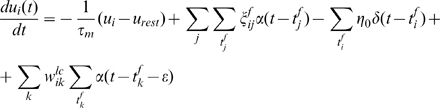
(11)where 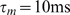 the membrane time constant, 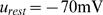 the resting potential, 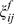 is a stochastic variable that takes the value 1 with probability 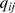 if the presynaptic place cell 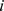 elicited a spike, and otherwise 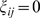, 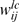 the synaptic strength of the lateral connections between neurons 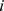 and 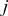, 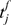 and 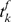 the spikes of the presynaptic neuron 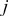 and 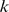 correspondingly, 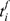 the postsynaptic spikes before time t and 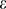 a small positive number. We note that the term 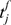 in the second term on the right-hand side refers to place cell firing whereas 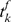 in the fourth term refers to action cell firing. We assume that the postsynaptic current is a short pulse:
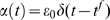
(12)with 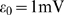 and 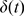 being the Dirac 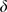 function. If neuron 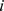 emits a spike, its membrane potential is reset by an amount 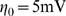. We note that with these definitions, our model is equivalent to a standard leaky integrate-and-fire model with pulse input and also a general case of the spike response model defined in Eq. (6).

In order to account for intrinsic noise or synaptic noise generated by additional presynaptic neurons that are not part of the model, we use a stochastic firing threshold [20],[63], also known as escape noise. Action potentials of the postsynaptic neuron 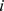 are generated by a point process with stochastic intensity 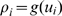 where 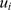 is an exponential function of the membrane potential [20],[64]

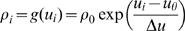
(13)where 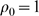/ms is the stochastic intensity at threshold, 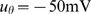 the formal firing threshold and 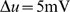 the width of the threshold region. We note that for the choice (13) the factor 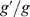 in the eligibility trace of Eq. (8) is a constant that can be absorbed in the learning rate. Unless stated otherwise, we use 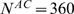 action cells for our simulations.

#### Lateral connections

The action neurons are connected in a ring with “Mexican hat”-type lateral connections. A weakly localized feedforward input to action cell 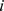 is sufficient, to cause within 25–200ms the formation of an activity blob. The location of the activity blob represents the next action of the rat. Because of the broad activity profile, not only the one neuron that is maximally active, but also neighboring active neurons can be reinforced during learning. For the sake of simplicity, we keep in our model the lateral connections fixed (i.e. they do not undergo synaptic plasticity) and use the equation:
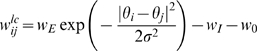
(14)with 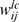 being the connection between neurons 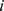 and 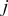, 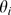 and 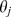 their corresponding preferred directions (the difference taken modulo 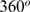), 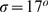, 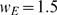 (weak connections) or 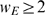 (strong connections) and 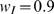. Local connections, i.e. 
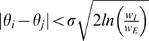
, are excitatory with 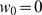 while connections over a longer distance are inhibitory, Eq. (14) with 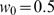.

We have chosen parameters such that blob formation takes place already at the beginning of the learning procedure. The effect of the lateral connections is similar to a Winner-Take-All mechanism.

#### Decision making

At each location in the maze, the rat has to choose the direction of its next move. The decision is taken after a bump-like activity profile has been formed in the action layer. We suppose that the population of action cells is modulated by inhibitory background input in the theta-frequency range. If inhibition is strong, no activity profile is formed and neurons are inactive. While background inhibition drops to zero an activity profile develops, centered around the action neurons with strongest feedforward input - and these represent the action the rat is going to choose next.

**Figure 3:**
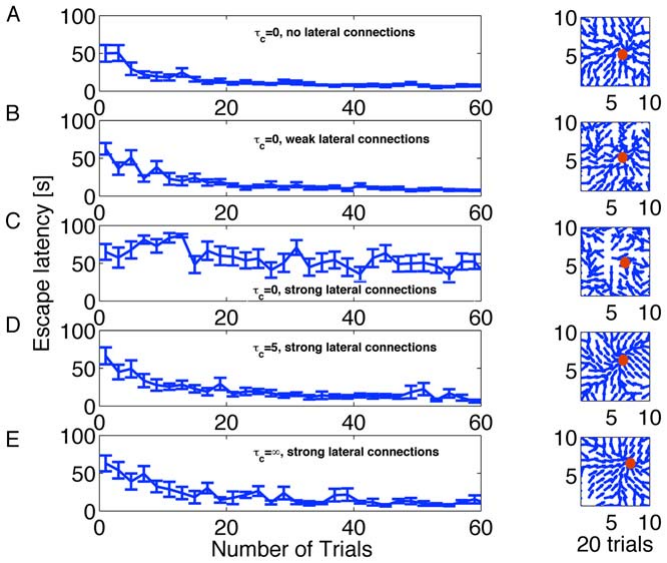
Learning performance for different variants of the learning rule. A. Left: Evolution of escape latency as a function of trials, without lateral connections (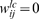) and 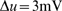. Right: Navigation map after 20 trials visualized in the water maze by a set of direction vectors. At each grid point (defined by the center of a place cell 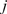) in the graph, we plot the normalized stochastic release probability 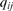 for fixed 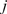 in the form of a population vector denoting the direction the animal would most likely take at this location. The red circle marks the position of the hidden platform. The navigation map is less smooth than with the standard choice of parameters of 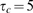
ms or 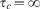, see D and E, Right. B. As in A with weak lateral connections, 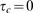 and 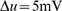. C. As in A with strong lateral connections, 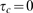 and 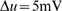. D. As in A with strong lateral connections, 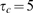
ms and 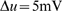. E. As in A with strong lateral connections, 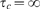 and 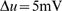. Initial release probabilities are set to 0.2; all other parameters as in Model architecture, Methods and Tables 1, 2.

In order to keep the model as simple as possible, we mimic the modulation of inhibition at theta-frequency algorithmically, by resetting every 200 milliseconds the activity of all action cells to zero. Otherwise, the dynamics is evolving freely according to the dynamical equations above. After 200 milliseconds, the rat takes its decision about the next action based on the population vector of the action cell firing rates. More specifically, the firing rate of action cells 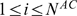 is estimated from a low-pass of the spiking activity
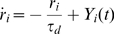
(15)where 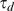 is a time constant set at 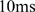 (or 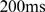) and 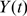 the entire postsynaptic train of the action cell defined as 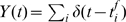, with 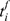 the 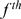 firing time of the 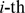 action cell. The direction that the rat will follow is described by the angle 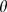 in an allocentric coordinate system, i.e. relative to room coordinates and calculated from the population vector:
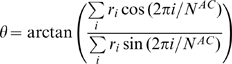
(16)where 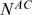 is the total number of action cells (typically 360 unless otherwise stated), and 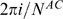 the direction of the 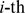 action cell. 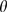 is calculated after a decision time 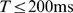. In Figure 3 C–E, T is the moment when the total activity of all action cells 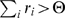, with 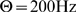, which is achieved if, e.g. 10 cells fire at more than 20Hz, a good indicator of when a decision (an activity bump) is formed. For all other simulations, 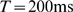, but in general any of these conditions are possible for each case.

### Watermaze performance

**Figure 4:**
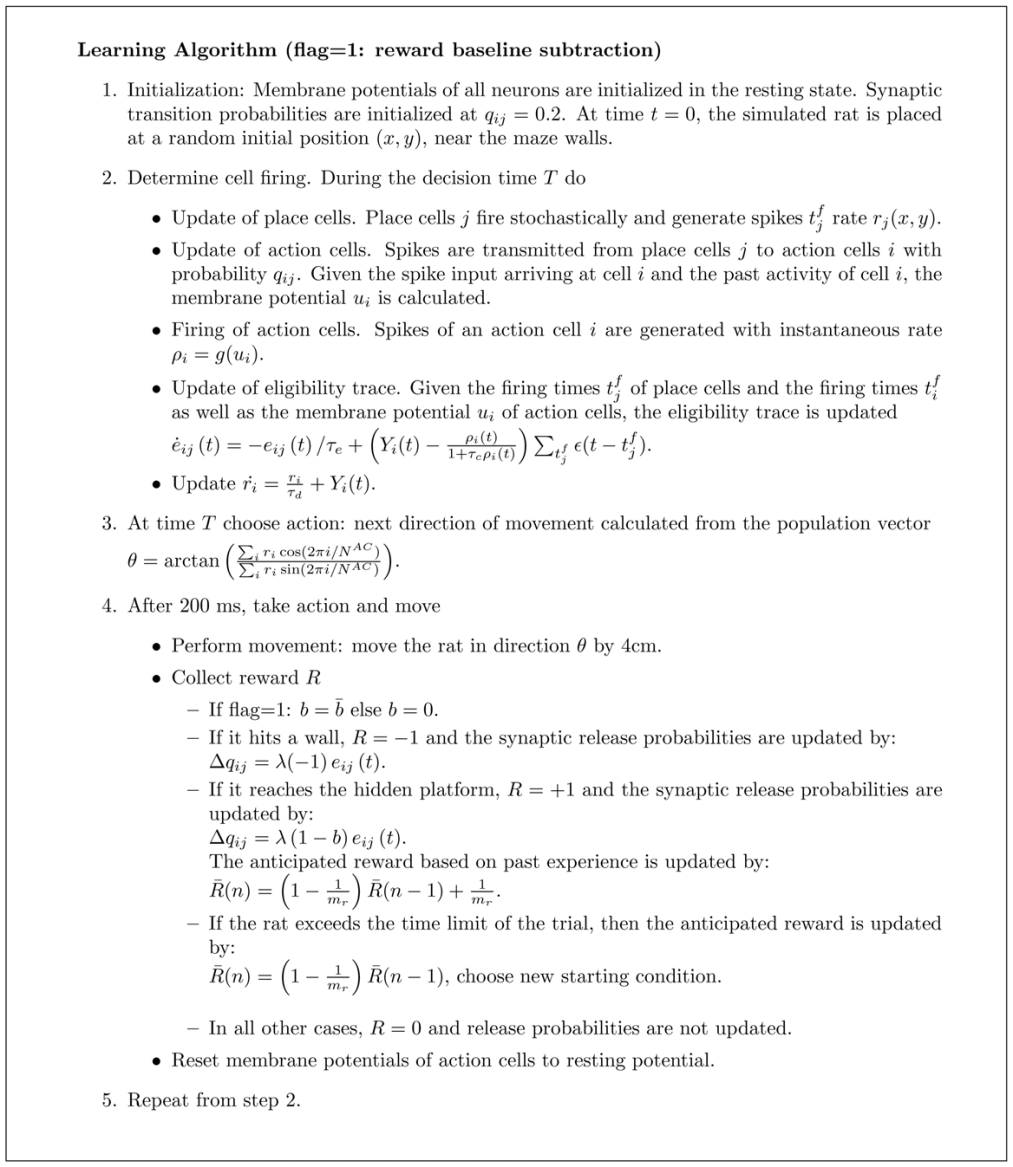
Learning algorithm. The decision time 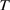 can be either 200ms, as in most cases, or can be based on a flexible criterion (Figure 3 C–E), see Results.

We perform simulations of a model rat navigating in a square maze of 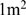, with a constant speed of 20cm/s. The rat performs a number of trials, with each trial consisting of an attempt to find the goal within a time limit of 90 seconds. At the beginning of each trial, the rat is placed near one of the walls of the maze. Actions are chosen at theta frequency (every 200ms). Between two action choices, the simulated rat moves by about 4cm. The rewarded position (target) is at a random position near the central region of the maze and remains fixed at the same position within a set of trials whereas the initial position of the rat varies, as in the experimental paradigm [51],[65],[66]. Positive reward 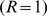 is only given if the rat reaches its target and negative reward 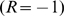 if it hits the wall. Thus, synaptic modifications take place either at the time the rat reaches the platform, 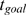, or at the time the rat hits a wall, 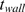. For an overview of the algorithm see Figure 4.

When a new *set of trials* starts, the positions of both the rat and the goal are reinitialized as well as the synaptic release of all plastic synapses in the model. Thus each new *set of trials* corresponds to a different animal.

#### Speed of learning

The performance of the rat is measured by the time it takes to reach the target, corresponding to the escape latency in the experimental literature [51],[65],[66]. In the panels of Figure 3 A–E we plot the escape latency versus trials for three values of the parameter 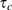 and three conditions of the mexican hat connections, zero (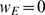, 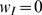 and 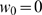), weak (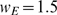, 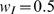 and 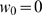) and strong (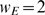, 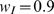 and 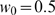). For zero or weak lateral connections learning takes place within 20 trials with any value of 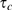 (Figure 3 A,B). The performance is similar to that seen in experimental data [51] and previous models [52],[55]. The standard deviation of the performance extracted from 10 repetitions of the learning experiment decreases while the task is learned.

Surprisingly, for lateral connections strong enough to form an an activity bump in the action cell layer, only the versions of the rule with a dominant Hebbian component (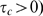 are able to learn the task (Figure 3 D,E), but not the standard policy gradient rule for spiking neurons (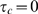, Figure 3 C). We believe that the critical parameter for a good performance of the policy gradient rule is neither the lateral connectivity nor the total input. Rather, it is a subtle interplay between the rule for the action choice (here: population vector based on firing rates) and the information encoded in the eligibility trace (see Discussion for more details).

In our model, actions depend on the population vector of the Action Cells calculated from the spike count about 200ms from each cell. Action cells, that have emitted most spikes, are most likely to dominate the action choice at a given place. Therefore, a standard Hebbian learning rule, that increases weights when pre- and postsynaptic neurons are jointly active, will set an eligibilty trace that is strongest for the action neurons that have most likely determined the action at this location. If that action led to a reward, those weights would be strengthened. Thus, it is not surprising that the model with 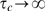 does work. What would be the situation for the standard policy gradient rule with 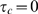
? As long as the expected number of spikes 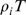 within the decision period of duration 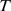 is smaller than one, the term 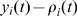 in the eligibility trace is positive for all neurons that have fired a spike – and these are exactly the neurons that determine the next action via the population vector. However, if the firing rates are higher, such a match between the memory kept in the eligibility trace and the chosen action is not guaranteed for in *single* trials of the standard policy gradient rule (see Discussion for more details). We report that the average instantaneous firing rate for the network without lateral connections, calculated as an average value among all action cells between the 20th and the 30th trial, is 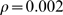 Spikes/ms. For the same network but with weak lateral connections is 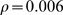 Spikes/ms (three times more) and with strong lateral connections an order of magnitude higher, i.e. 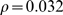 Spikes/ms. More importantly, the neurons inside the activity bump fire in Figure 3 C–D at a rate of 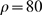 Hz yielding 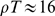 spikes, 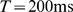. Thus, the eligibility trace of the most active synapses accumulates about 16 spikes of the postsynaptic neuron.

For the case of 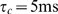 we compared the situation without baseline subtraction 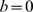 and with a baseline subtraction 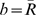, and the results are similar (data not shown). However, if we follow learning for more than 100 trials, the factor 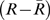 increases long-term stability, as expected.

#### Navigation map

**Figure 5:**
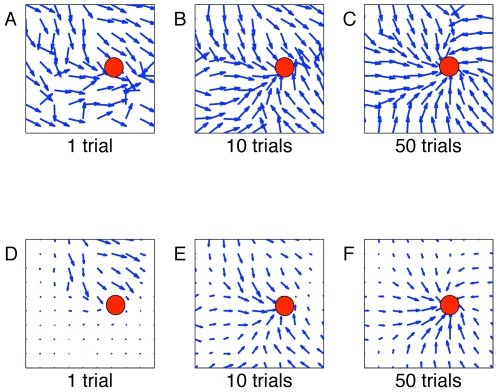
Navigation map of the rat visualized in the water maze by a set of direction vectors, for 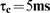. Panel A depicts the map formation after 1 trial, B after 10 trials and C after 50 trials. The simulated animal has developed a rough strategy to reach its goal already within 10 trials. For details on how the navigation map is calculated, see Figure 3. Learning rate decays as a function of mean reward. Preferred directions are plotted as normalized vectors. In D–F we plot the same navigation maps with non normalized vectors. While F seems to contain no information about preferred directions near the wall (due to scaling of arrows), the normalized version C confirms that the simulated animal has developed a strategy for all positions in the maze.

**Figure 6:**
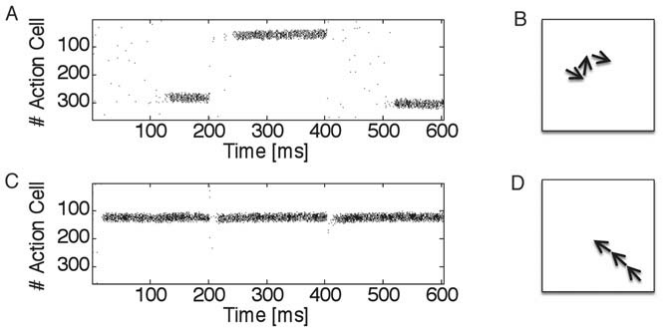
Sequential formation of actions. Spiking activity (dots) of the population of action cells as a function of time during three theta-cycles. A: Before learning, the moves of the simulated animal reflect random exploration of the space leading to a B: discontinuous trajectory. C: After learning, the three consecutive actions exhibit similar direction choices leading to D: a continuous movement.

Given the rat's location, the direction of the next move is decided by the population vector of the action cells. Suppose that the rat is in the center of the place field of cell 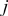. Then the population activity of the action cells is, to a large degree, controlled by the strength of the synapses connecting place cell 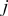 to the different action cells: the stronger the synaptic weight 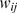 to an action cell 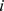, the more likely that the action represented by 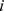 would be chosen. We therefore use the population vector of the synaptic strength of the feedforward connections from a given place cell to visualize the direction of motion starting at that location. The combination of vectors gives a flow map, corresponding to the navigation map of the rat. In Figure 3 A–E right hand side we show the navigation map after the 20th trial for different 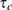 values and lateral connections. It is noteworthy that the quality of the navigation map is increased under the presence of strong connections (and 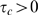). Figure 5 shows the evolution of the navigation map of the rat for 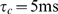 after 1, 10 and 50 and 100, with A–C depicting preferred directions as normalized vectors and D–F as non-normalized vectors. A–C show that already within 10 trials the simulated animal has developed a strategy for reaching the goal, and D–F show the relative strength of the population activity, which increases as the animal moves closer to the target. Adequate learning has been achieved, if for any starting condition the flow is towards the target zone. We find that already after 10 trials, a rough strategy for the Morris watermaze task has been developed, which is refined during subsequent trials. Figure 6 confirms that trajectories become smoother during learning. A sequence of 3 action choices has a strong random component at the beginning but is nearly continuous after 100 trials.

#### Performance vs number of place and action cells

**Figure 7:**
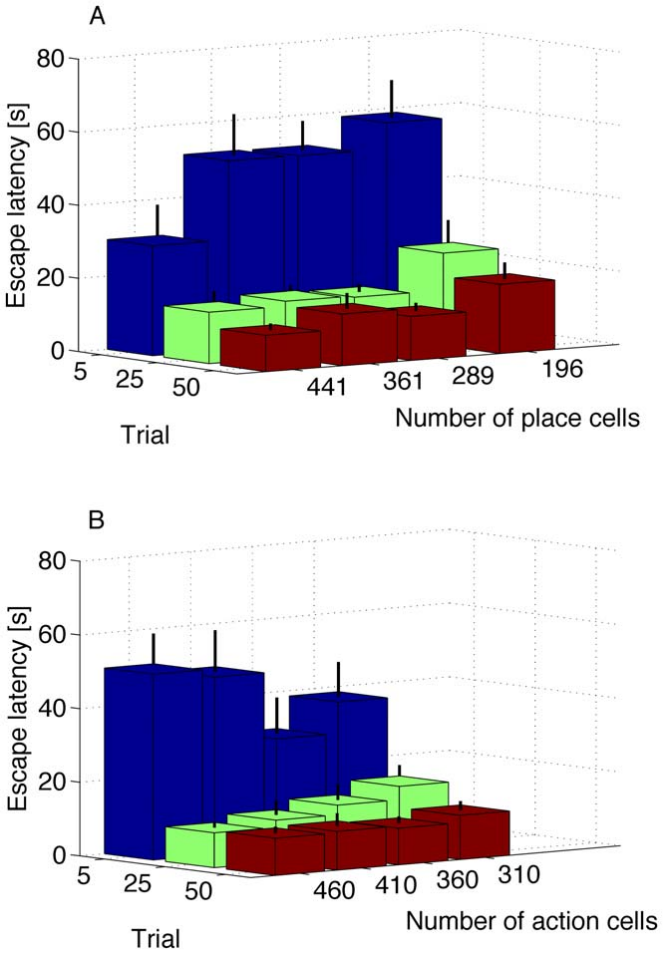
Scaling properties of the network. A: Average time it takes the rat to reach the hidden platform at the 5th, 25th and 50th trial versus number of place cells. B: Average time it takes the rat to reach the hidden platform at the 5th, 25th and 50th trial versus number of action cells. Error bars show standard error for the mean. Note the improvement as the number of place cells is increased. This is due to the systematic formation of an activity bump in the presence of stronger input. The same parameters were used in producing all sets of these simulations: 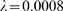, 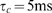, 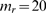, 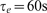, 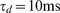, 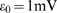, 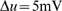, 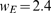, 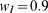 and 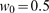, see also Results. For B, place cells are located every 5cm, with a gaussian receptive field of 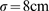, and maximum firing rate 120Hz. To reduce CPU time, for this set of simulations we do not implement the stochastic release.

How does the performance depend on the number of place and action cells? For place cells, we require that the surface of the water maze will be sufficiently covered by neurons with overlapping receptive fields. This continuous space representation (due to overlapping receptive fields) leads to simultaneous learning of nearby neurons, resulting in no significant change in performance even when doubling the number of neurons in each dimension, see Figure 7 left. Similarly, a minimum number of action cells is required such that the activity profile will be created, but increasing the number of cells beyond 300 cells or so does not change the performance. The reason is that the activity profile has always roughly the same width (about 30 degrees) in action space. Adding more cells just increases the number of cells in the activity bump. In Figure 7 right we plot the average time it takes the rat to reach the hidden platform at the 5th, 25th and 50th trial versus number of action cells. We note that the performance does not significantly change. This is in contrast to standard reinforcement learning in discrete state and action spaces where increasing the number of states or actions increases the number of free parameters, so that learning becomes slower [1].

## Discussion

We presented a spike-based reinforcement rule which combines a global reward signal with two local factors available at the site of the synapse. The first local component is a contribution generated by presynaptic spike arrival and enters the update rule in the form of the EPSP. The second local component depends positively on postsynaptic spike firing and negatively on the postsynaptic membrane potential. The relevance of the membrane potential decreases with 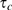 and vanishes for 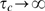. The third factor of the learning rule is the global reward signal that can be associated with neuromodulators such as dopamine [22]. Thus the eligibility trace which combines the two local factors marks the synapse that can undergo LTP or LTD. The actual weight change is implemented only after confirmation by a global reward signal that may arise with a significant delay. Such a picture has interesting relations to the model of synaptic tagging and capture [27] where synaptic connections undergo preliminary changes into early LTP or LTD that decay unless they are stabilized if plasticity related protein is available. Synthesis of these plasticity related protein can occur with a delay and requires neuromodulators such as dopamine [28],[61].

### Global factors, neuromodulators, and TD-learning

In the introduction we mentioned two classes of theoretical reinforcement learning algorithms, that is, temporal difference (TD) learning methods on one side [1],[43] and policy gradient methods on the other side [39],[40]. Our model task and model architecture would allow to test both types of algorithm in the form of a three-factor rule (see [45], [52]–[54] for examples of a TD algorithm for this task). One major difference between the TD algorithms and the algorithm in this paper lies in how the global factor encodes neuromodulatory feedback about the reward. In the case of TD-learning, the global factor expresses the difference between the reward received and the expected reward (where the expected reward is calculated from the temporal difference between reward expectations of subsequent states [1]), whereas in the case of the gradient learning algorithm of this paper the global factor correspond to reward itself, possibly after subtraction of a baseline. Here we used a variant of the idea of a baseline, since we subtracted the mean reward averaged over order 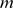 previous trials, see also [41]. Subtracting the expected reward should help rapid re-learning in case of the change of the learning task (e.g., by moving the escape platform to a different location) [67]. Similar to TD learning the global factor can be interpreted in this case as reward minus expected reward. In contrast to TD learning, the expected reward arises from a running average, rather than a difference in reward expectation across different states as in spike-based TD algorithms [37],[45]. Experiments on dopaminergic neurons suggest that the phasic dopamine signal indeed encodes a TD-like error signal [22] although other interpretations of the dopamine signal [68] and the involvement of other neuromodulators is also possible [69].

Our spike-based navigation model features a continuous description of state and action. Unlike traditional TD models with discrete state and action space, increasing the number of neurons while keeping the width of place fields and the width of lateral interactions between action cells constant) does not change the performance of our model. In addition, the model provides insight in studying decision making in the context of navigation. We hypothesized that activity is modulated at theta frequency. Note that we implemented an extreme situation where the action choice is taken at the end of each theta cycle. However, it is easily possible to have the rat take an action as soon as the activity profile is formed. The time necessary to create an activity profile determines then a minimal time for deciding a new action. If this is so, then our model predicts that the time it takes to choose the next action is much faster after learning than before learning, because activity profiles are more rapidly formed with strong feedforward input - as it would occur after learning.

### Morris water maze task

To test the potential of our spike-based reinforcement rule, we have applied it to a biologically relevant navigation problem, i.e., the Morris water maze task with variable start condition [51]. Our model which is based on a simplified model of place cells and action cells reproduces behavioral data of real rats in terms of escape latency versus learning time. The model consists of about 700 spiking neurons, in two layers and includes both feedforward and lateral connections. In the first trial, the model rat moves in a random trajectory and finds the hidden platform by exploration. Across several trials, approach paths towards the platform are reinforced, so that the escape latency is reduced.

A positive reward is delivered when the model rat reaches the target location. In the model, we also use negative reward at the boundaries of the maze so that the simulated rat will learn to avoid the walls. This aspect does not reflect the fact that, normally, during development (or even because of reflexes present at birth) we could assume that the rat already knows how to avoid obstacles prior to the start of the watermaze task. However, since we did not want to include into the model prior knowledge about obstacle avoidance, we let the simulated rat ‘discover’ the effect of the walls. Since our model assumes the existence of place cells, we must assume, however, that the rat has had some pre-exposure to the environment long enough to establish place fields. Experiments have shown that place fields are established during a first exploration of the environment, so that during the learning task, they can be considered as given. Moreover, typical experiments require prior habituation of the animal to the environment, so that place cells may be formed. A model where place cells are learned from visual input and path integration is also possible [53].

While in our model place cells can be easily linked to cells in hippocampus, a direct identification of the action cells with the biological substrate is more problematic. In rodents, navigation in water maze task involves two competing pathways [70]–[72]. The first one is involved in taxon navigation (e.g., approaching a visible target, which could be achieved with stimulus-response habits [73] also called response learning [71]) and associates visual input directly with motor actions. It is independent of hippocampus and the action choice for this navigation strategy can presumably be linked to the the dorsal striatum of the basal ganglia (caudate-putamen in the rat). The second one is concerned with locale navigation (also called place learning [71] or cognitive map [74]) and this is the relevant pathway in the context of the present model. It relies on hippocampus [51],[70],[71] where the activity of place cells presumably encodes the location of simulated animal. The choice of motor actions is presumably encoded in the nucleus accumbens (NA) of the ventral striatum where our hypothetical action cells could be located. The Mexican hat connectivity between action cells is a simplification of a more complex wiring scheme, where excitatory neurons project to inhibitory neurons, which in turn inhibit other action cells that encode for “different” directions, see for example a biologically plausible winner-take-all [75]. However, to reduce the connectivity in our network, we chose to simulate the equivalent but simpler Mexican hat scheme.

One limitation of the model is that learning only takes place in the presence of a reward signal with the consequence that learning can only occur in a limited radius around a reward. The radius is related to the time scale of the eligibility trace, governed by the time scale 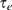. In a large environment where at a fixed speed 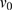 it takes much longer than 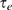 to traverse the environment, information about the target falls off exponentially with a spatial scale 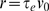. In our case we would encounter this limit only if the environment were scaled by a factor significantly larger than two.

In a TD framework, the situation would be different: even without an eligibility trace, information about the presence of the reward can slowly diffuse across the landscape of estimated reward expectation values 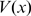 where 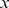 is the position, even beyond the radius 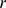 discussed above. This slow diffusion of reward information is possible because the update is not proportional to the reward itself, but to a factor 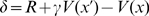 where 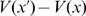 gives the difference between the reward estimation at location 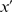 and that of the previous location 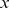 and 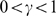 is the discount factor. An implementation of a TD learning structure in spiking neurons is possible using the actor-critic scheme [37],[45]. If a TD algorithm is implemented in discrete time with time steps 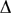, and if the rat runs as before at a constant speed 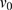, the distance travelled between two time steps is 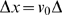. After convergence, the value function decreases exponentially with the distance from the target on a lenght scale 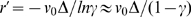. (In other words, once the exponentially decaying 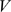 dependence is reached, the 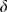 in the update rule vanishes). A comparison with the result in the previous paragraph shows that the time scale 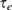 of the eligibility trace in our model plays a role similar to 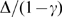 in the TD model. The role of the eligibility trace has been extensively discussed in [35]; in our interpretation the eligibility trace is implemented in the synapse and its time constant 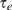 corresponds to the decay time of some biochemical substance.

The parameter 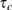 is an ad-hoc parameter that allows us to vary the behavior of the learning rule from pure Hebbian to optimal in the sense of policy gradient theory. We do not wish to explicitly associate it with a biological substrate, but in our model it would be closely related to the voltage dependence of LTD.

Recently, the influence of neuromodulators on spike-timing dependent synaptic plasticity has been investigated in a small number of studies [31],[76]. These studies show that dopamine acts on the temporal profile of STDP, rather than a simple scaling of STDP. This result is in contrast to some of the assumptions of standard reward-modulated STDP [35],[36], but also in disagreement with policy gradient rules [33],[34],[38] and the learning rule discussed in this paper. For plasticity in the cortico-striatal synapse [31], but not for glutamatergic synapses in hippocampal neurons [76], dopamine is necessary for synaptic plasticity. In other words, learning is gated by the presence of dopamine. The plasticity rule in the cortico-striatal synapse is in that respect similar to the reward-gated plasticity rules in the present paper. Interestingly, the striatum is potentially involved in action selection.

It should be noted that in standard cortical STDP experiments [77],[78] the level of dopamine and other neuromodulators is not explicitly controlled and a background level of dopamine cannot be excluded. Therefore, it is unclear whether cortical STDP is unsupervised or shows a, possibly weak, dependence upon neuromodulators.

### Limitations of policy gradient methods

An important parameter in our family of learning rules is the parameter 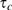, that tunes the learning rate such that for neurons that fire at high learning rates LTD is reduced. To see this, consider an instantaneous firing rate 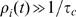. Then the term 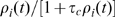 converges to 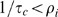. Hence, the decrease of the eligibility trace in the absence of spikes is limited. Note that because of 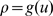 high rates correspond to large depolarizations of the membrane potential. For 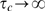, the term 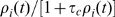 vanishes, and the membrane potential 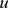 no longer enters the update of the eligibility trace. In this case the eligibility trace pick up Hebbian correlations 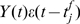 between EPSPs caused by presynaptic spike arrival and postsynpatic firing.

The case 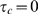 corresponds to the learning rule derived from the reward maximization as shown in the methods section, i.e., 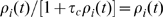. For 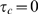 the two postsynaptic terms, i.e., spike firing and voltage dependence cancel each other *on average*, because spikes are generated with the stochastic intensity 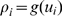, hence 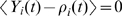 where angular brackets denote expectation values. However, a specific realisation of a spike train (e.g., one with more spikes than expected) may lead to a reward whereas another one (with less spikes than expected) does not. In this case only the rewarded one is learned, making it more likely that the same spike train is reproduced again for the same input [34]. In fact, a large class of learning rules for conditioning can be explained as a reinforcement of the covariance between reward and a noise-induced variation of the output [79].

There are three reasons why the standard policy gradient rule with 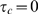 derived from reward maximization is not applicable in our scenario.

(i) Large learning rate. The learning rule derived from reward optimization is a batch rule, i.e., it assumes averaging across several realisations and many inputs. For the transition to the online rule we had to assume a very small learning rate so as to make the learning self-averaging. If learning is slow, then thousands of trials are needed before the weights change significantly, so that online and batch have nearly the same effect.

**Figure 8:**
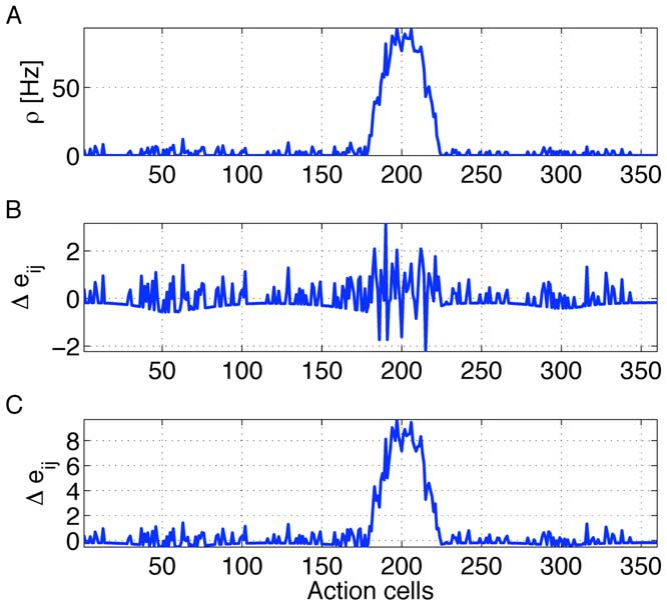
Action cell activity and eligibility trace. A: Snapshot of mean firing rate of action cells during one of the trials while the simulated rat is in the center of the place field of cell 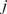. The chosen action is a movement in direction 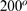. B and C. At this instance, the momentary value of the eligibility 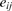 is plotted as a function of 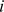 for fixed 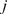 (fixed presynaptic location). B: For the rule with 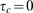 the profile of eligibility traces is stochastic with zero mean and maximum variance inside the activity bump. C: For 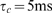 the profile of eligibility traces reflects the activity profile shown in A.

In order to explain biological learning paradigms, we need, however, to achieve learning after as few as ten trials. If we work with a large learning rate 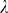, then terms of the form 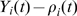 that average away in the batch rule, can make a big contribution in the eligibility trace of each single trial and can cause weight changes that are not causally linked to the reward. Thus the eligibility trace encodes noise, rather than relevant correlations. With small learning rate, these correlations would average away (and only those systematically linked to the reward would survive), but with a big learning rate these changes act like a diffusion process. Moreover, the effect of the diffusion increases with the number of spikes in the decision window and therefore is highest for neurons having a large firing rate 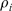. Large firing rates 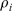 appear in particular after learning for neurons inside the activity bump, because strong lateral input is added to strong feedforward input. Hence the eligibility trace is most noisy in the center of the bump, as shown in Figure 8 B.

(ii) Decision by firing rates, not by spikes. The close relation between reward-maximisation by policy gradient rules and supervised learning shows that the spike-based rule with 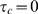 is optimal to learn a specific spatio-temporal spike pattern [34]. However, what counts for the action choice in our simulations is the firing rate accumulated over 200ms. To understand the importance of this distinction let us consider two Poisson neurons coding for actions ‘left’ and ‘right’, respectively. The action ‘right’ is the rewarded one. Suppose the neurons receive inputs that drives the neurons coding for ‘left’ at an intensity 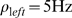 and the other at 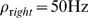. Suppose, because of intrinsic noise, the neuron coding for ‘left’ fires 2 spikes in a decision interval of 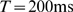, while the neuron coding for ‘right’ fires 9 spikes in the same time interval. If actions are chosen according to maximal firing rates, the neuron coding for right wins, the system performs the ‘right’ action and receives reward. However, the term 
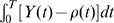
 is negative for the neuron coding for ‘right’ and ‘positive’ for the neuron coding for ‘left’. Hence, after reward is received action ‘right’ is weakened, while action ‘left’ is reinforced, in contradiction to the fact that action ‘right’ is the correct one that should be reinforced. To put it differently, action neurons have to learn that (a) precise spike timing is irrelevant and that (b) even the absolute rates are irrelevant because all that matters is the firing rate relative to those of the other neurons. Since the policy gradient rule is desigend to learn precise spatio-temporal spike patterns, it is not ideally suited for our paradigm. In contrast, reward-modulated Hebbian learning just make the neurons that fired at high rate (and influenced the action) fire at even higher rates. In the specific task we are considering this happens to be a viable strategy.

(iii) Populations of neurons, not single neurons. Furthermore, because of the formation of an activity bump and the readout by a population vector the decision about actions is taken by a *population* of neurons rather than individual neurons. Learning in populations suffers from the problem that firing of individual neurons may differ from the majority vote that led to the actions, so that giving appropriate feedback is nontrivial [80].

Figure 8 illustrates the detrimental interaction of points (i)–(iii) for the standard policy gradient rule. We focus on a presynaptic neuron 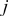 which codes for the current location of the rat so that synapses from 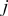 to all action neurons 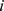 are active. The instantaneous firing rate 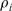 represents the activity bump (Figure 8 A). Despite the fact that the term 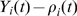 has an expectation value of zero, the term 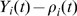 gives a non-neglibible contribution in each trial, see also Figure 1 C – as it should be since policy gradient rules need to exploit fluctuations. However, we would like to emphasize two aspects. First, the standard deviation of 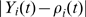 grows with time, similar to a diffusion process. Second the diffusion constant increases with the instantaneous rate 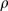. Therefore the deviation from the expected value 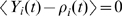 increases with the expected number of spikes 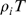 the neuron emits during the decision interval of length 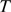. The eligibility trace is sensitive to this deviation. In the case of our action learning model, the consequence of the above argument is that the set of significantly positive eligibility traces 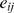 for fixed presynaptic neuron 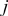 includes not just action neurons within the activity bump, but also those representing other directions; see Figure 8 B. Moreover, the variation of eligibility traces between neighboring neurons inside the activity bump is big, because the expected number of spikes is higher for neurons inside the activity bump. In particular, several synapses from a fixed presynaptic neuron onto neurons in the bump have eligibility traces that are significantly negative (corresponding to the fact that some neurons in the bump fire less spikes than expected from the firing rate 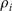, see point (ii) above). This leads to the problem that eligibility traces of individual neurons do not reflect the action choice represented by the population of active neurons [80]. Simply speaking, neurons inside the bump are those that determine the action even though their eligibity trace can be negative.

The parameter 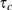 in our learning rule gives a systematic positive bias of the postsynaptic term for those postsynaptic neurons that have a large firing rate. Thus the eligibity trace is maximal for neurons within the bump of activity, i.e. for those representing the action that is actually chosen; see Figure 8C. Hence, if the sequence of actions leads to a reward later on, the synpatic weights between those presynaptic place cells and postsynaptic action cells that actually led to the sequence of actions are maximally strengthened. Because of the bounds on the weight dynamics, these weights will eventually converge towards a release probability of 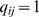. We note that all neurons outside that activity bump have very low activity, so that 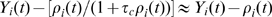 has a zero average and only small fluctuations. Hence, a learning rule with 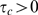 is expected to work better in the case of large learning rates 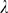, and high firing rates 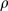, and a decision criterion based on a population vector calculated over a long time period.

In a general spike-based learning problem where the aim is to learn a spatio-temporal spike pattern, the high variability of eligibility traces would allow to explore a large space of firing patterns. However, in our case with lateral interactions and decisions based not on detailed firing patterns, but only on population vector data integated over 200ms, the bias towards high activities identifies neurons in the bump that participate in the action choice.

Indeed, a learning rule with 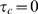 does work in the situation where (a) there are no lateral interactions between the action cells or (b) decisions are based on less than one spike per neuron on average. In the latter case, every spike is unexpected, and basing a decision on the population vector chooses an action that is indeed caused by a fluctuation.

In principle four action neurons would be sufficient to encode the direction of the next action (e.g., [45],[53]). In this case, learning rules based on either policy gradient [45] or naive Hebb [53] work. However, it is likely that in biological brains actions are encoded by large populations of neurons. In order to achieve fast learning despite a large population of action neurons, action neurons must share information during learning – and this can be achieved by the formation of activity bumps. The results of this paper show that in the presence of activity bumps and population vector read-out based on spike counts, the spike based policy gradient rule no longer works, whereas a rule with a bias towards Hebbian correlation does.

From a technical point of view, neither stochastic synapses nor voltage dependent plasticity is critical for the function of the model, however they are both desirable properties for the biophysical plausibility of the rule. In our model, the stochastic release probability of the synapses is hard-bounded in order to maintain reasonable values, for a biophysical implementation of such bounds see [46].

Also a reset it is not necessary to take place exactly every 200msec; in principle may occur at any point that the activity bump is formed. We require to reset the activity in the action neurons layer only (or equivalently we could clamp the AC activity for say 10ms) so that the activity profile will not become “sticky”, but in no other way the learning would be affected. Without reset, the rat will end up again learning the position of the platform, but its movements will become more curved. A negative input would be desirable after a decision is formed so that at the beginning of the learning the next action will not depend on the previous one. This negative input may arrive at any point after a decision (activity bump) has been formed. We chose 200ms so that this could coincide with the theta rhythms, but it could have been 150ms or 300ms, or a random interval (as we demonstrate in simulations).

## Methods

Policy gradient methods [39],[40] have been applied to spiking neurons several times and result in spike-based formulations of reward-based learning [32]–[34],[38]. In the following subsection we derive again the same rule, but with the aim to show that the derivation holds even in a network of spiking neurons with strong lateral connectivity (see also a comment in [40]). In the following two subsection we make the transition to an online formulation with eligibility traces and stochastic synaptic transmission. In subsection we leave the policy gradient framework by introducing the parameter 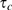 in order to enable a smooth transition between the standard policy gradient rule and a naive Hebbian rule that measures directly correlations between presynaptic spike arrival and postsynaptic firing on the time scale of the EPSP. The rule used in the main body of the paper is a mixture between policy gradient and naive Hebbian rules.

### Derivation of the learning rule

To derive a learning rule for a highly connected network with action cells 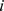 with lateral connections receiving from input from place cells 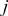, we shall first consider a restricted scenario where the rat always starts a trial in the same initial location and is left to move around for a fixed duration 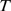. We shall denote by 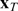 (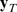) the spatio-temporal spike pattern generated during this time by all place (action) cells. The reward, administered at the end of each trial, depends on the trajectory of the rat in the water maze. Given the fixed initial location, this trajectory is determined by the firings of the action cells. So we write reward as a function 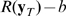, where b is the reinforcement baseline [39], without explictly noting the dependence on the initial position of the rat. Expected reward then is [32],[34]

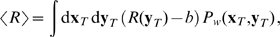
(17)here 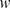 denote the strengths of the synapses connecting the action to the place cells, and 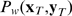 is the probability that the network generates the total spike pattern 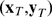.

In our model 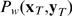 can be decomposed as (see also Decomposition of probability):
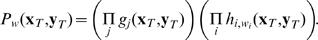
(18)Here 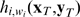 is the function giving for the action cell 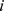 the single neuron probability that it generates its spike train 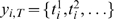 with an input consisting of all the other spikes produced by the network. Similarly, 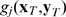 is the single neuron probability function for the spike train produced by the 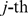 place cell given its input (determined by the other spikes in the network).

Note that the above product form does not imply that the spike trains are statistically independent. This is obviously not the case: First, due to the lateral connections between the action cells, and, more importantly, due to the simple fact that the action cells decide on the rats trajectory and thus influence the firing of the place cells. The product form simply represents the fact that the internal stochastic processes which modulate the translation of presynaptic input to postsynaptic output are assumed to be independent between different cells. In other words, *given* the input spikes from all other neurons and its own previous spikes up to time 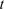, the neuron 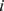 decides locally whether it fires between 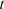 and 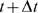 or not (i.e., we activate an independent random process for each neuron in each time step of the simulation), see section Decomposition of probability.

An explicit form for 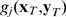 would be rather complicated, due to the involved calculations mapping the action cell firings to the trajectory of the rat. Luckily, we just explicitly need 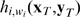. Note, and this is in fact the crucial feature of the decomposition, that 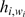 does not depend on all feed-forward weights, but only on the weight vector 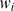 of the synapses actually projecting onto neuron 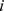.

To calculate the gradient of the expected reward (17), we first rewrite the probability 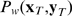 as
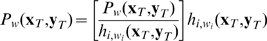
(19)and note that in view of (18) the term in square brackets in fact does not depend on 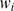 (even if this is not apparent from the notation). Now, for the synapse connecting place cell 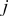 to action cell 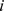 the gradient calculation is
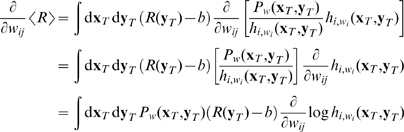
(20)The last line yields a batch rule for synaptic changes. We first average
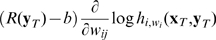
(21)over many trials and then use the result to update the synaptic strength. The biologically reasonable online version of this is to already update after each single trial, i.e.
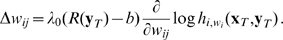
(22)Often we replace the reinforcement baseline 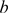 with the estimate of upcoming reinforcement based on past experience 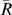 [39]. In the context of on-line learning, our initial requirement of a fixed initial position is no longer necessary since we calculate the expected reward by averaging not just over trials with the same but also over trials with different initial positions.

The crucial element of the learning rule is the conditional probability of creating certain outputs 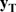 (and hence taking certain actions) given an input 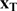. In order to calculate the conditional probability 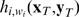 that neuron 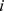 fires a spike given the past, we need to introduce a neuronal model. Following the approach of Pfister et al [34], we assume that neuronal activity can be described by the Spike Response Model (SRM) [20]:
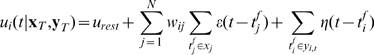
(23)where 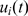 is the membrane potential of the neuron 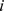, 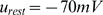 is the resting potential, 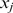 is the set of postsynaptic spikes, 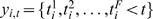 is the set of postsynaptic spikes up to time 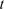, 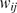 the synaptic strength between the presynaptic neuron 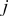 and the postsynaptic neuron 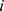, 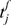 is the 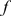
th firing time of the presynaptic neuron 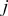 and 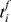 the 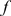
th firing time of the postsynaptic neuron 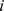. The sum is restricted to firing times before time 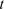. The kernel 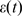 describes the time course on an excitatory postsynaptic potential (EPSP) and 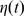 the spike-afterpotential. We would like to emphasize that for an exponential kernel 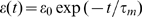 and exponential spike- afterpotential 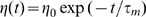, the SRM becomes identical to a leaky integrate-and-fire model with membrane time constant 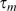 [20] as used in Eq. (11) in the results section.

Given a membrane potential 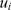, action potentials are generated by a point process with stochastic intensity 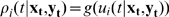, where 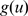 is some positive nonlinear function. To be specific, we take an exponential function
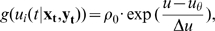
(24)where 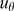 the formal firing threshold, and 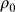, 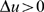 parameters. Thus the higher the membrane potential, the more likely is the neuron model to fire.

With the above neuron model, the probability of neuron 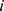 to emit a particular set of postsynaptic spikes 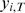 in the period 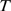 given the input 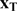 and 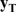 from all neurons in the network except neuron 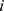 is given by:
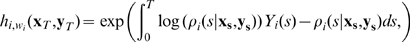
(25)with 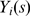 representing the postsynaptic spike train of the neuron 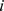 up to time 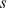 as a sum of the Dirac 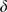 functions, i.e 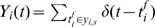. Taking the partial derivative in respect to the synaptic weight 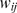, we have the following equation [34]:
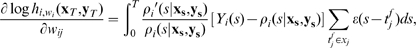
(26)where 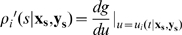, 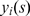 being the set of postsynaptic spikes that occurred before 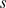, and 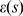 the EPSP kernel. Note that for the exponential function 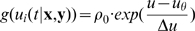, we have 
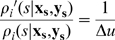
, so the learning rule becomes:
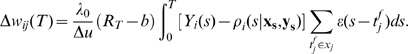
(27)Here 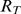 is the total reward received during or after a trial of total duration 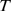.

### Eligibility trace

In order to illustrate the mathematical structure of Eq. (27), we consider the time point 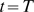 at the end of the trial and integrate backwards in time
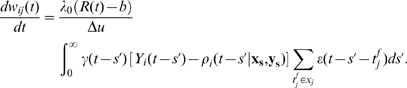
(28)where 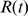 is the momentary reward at time 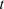. Here 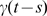 is a weighting function that allows us to give different weights to events in the past. If we take 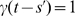 for 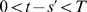 and zero otherwise, and evaluate at time point 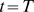, we retrieve exactly Eq. (27) under the assumption that the reward is given according to one of the following two schedules: (a) all the reward 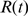 is delivered at time 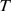, i.e., 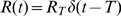 and a negative 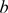 is applied at every time step; this is the scenario we have in mind with our notation 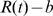 that we use throughout the rest of the methods section, since it simplifies the development of the theory. Or, (b) no reward is given in the interval 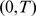 and an effective reward 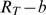 is applied at time 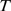, i.e., 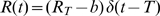. This is the scenario we used in the simulations in the main body of the paper. The baseline is either 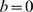 or 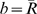.

Starting from the interpretation (a) we can turn to an online rule in continuous time where rewards can be delivered at arbitrary moments. To arrive at a more elegant representation of the rule, we replace the step function 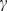 by an exponential kernel 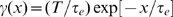 for 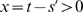 and zero otherwise. Then we have
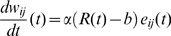
(29)

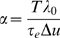
 is a learning rate and 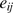 is called an eligibility trace [1],[32]. For our specific model we have
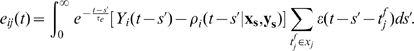
(30)


Because of the exponential in the integral the eligibility trace can be rewritten as a differential equation
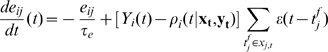
(31)


### Stochastic versus continuous synapses

We consider stochastic binary synapses 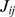 with 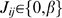. Synaptic transmission is stochastic with a release probability 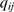. Learning affects the release property so that increasing the weight 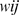 of the synapse by the above update rule will increase the release probability. We choose proportionality factors so that the expectation of the binary synaptic transmission over time is equal to the continuous synaptic weight 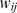, i.e. 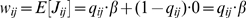. and thus, with 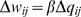, we have for binary synapses instead of Eq. 29 the following learning rule
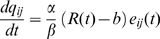
(32)We impose a hard bound 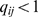 that reflect the interpretation of 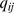 as a probability of transmitter release. In order to guarantee sufficient exploration, we also impose a non-zero lower bound 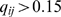


The factor 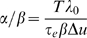 can be absorbed by a learning rate 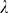 yielding the final online-rule
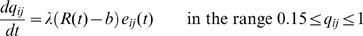
(33)

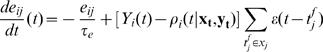
We note the typical structure of a three-factor learning rule. The eligibility trace picks up correlations between EPSPs 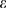 caused by presynaptic spike arrivals 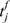 and postsynaptic firing times 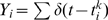 as in a STDP learning rule [34] which is then combined with the reward signal [33]–[35].

### From a single rule to a family of rules

We extended our rule by introducing *ad hoc* a variant with a parameter 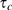:
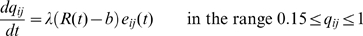
(34)

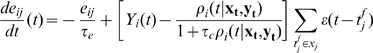
In the limit of 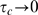 this reduces to the rule derived above.

Eq. (34) in discrete form becomes:
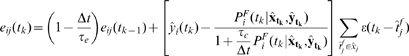
(35)with 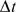 being the time step, 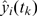 being 1 if a spike is emitted in the interval 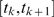 and 0 otherwise and the hat (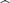) operator denoting discrete firing times. The quantity 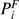 is the probability that the postsynaptic neuron emits a spike in the interval 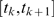 given the input spike trains (denoted 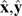 in discrete time) and is computed as
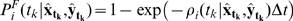
(36)which computationally advantageous for large timesteps, see also [20].

In Figure 1 we plot the factor
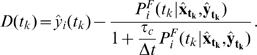
(37)The voltage trace is obtained by integrating Eq. (11) for constant input, i.e. presynaptic spike arrival is replaced by a positive constant.

### Relationship to other rules

Interestingly the rule developed by [34] as well as the variation presented here can be mapped to Associative Reward Inaction (ARI) [39],[81] in discrete time. With Eq. (27), and ignoring the baseline subtraction, we have
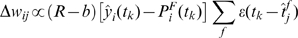
(38)


Let us assume a rectangular EPSP of duration of one time step and unit amplitude. Hence, the EPSP 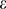 can be replace by a binary variable 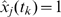 if a spike has arrived at the synapse j at time 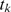, and with 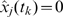 in the absence of a spike. We then have:
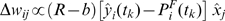
(39)We note that according to the above derivation 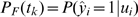 is a sigmoidal function of the membrane potential 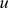. Hence, dropping the hats (that we used to denote discrete time) we have exactly the update rule of the ARI:
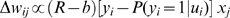
(40)Similarly the learning rules of [32],[33] also correspond to ARI or its modern forms of policy gradient. In fact the rule in [33] is derived from the framework of [40]. The rule of [32] is a special case of the rules by [33],[34], since it makes use of a memoryless Poisson neural model, wheres our derivation here includes refractoriness via the kernel 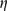.

### Decomposition of probability

Here we show that the probability 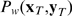 of the place cell spike pattern 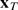 and the action cell spike pattern 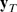 to occur can be decomposed into the product
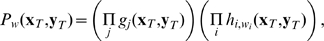
(41)as mentioned in the Methods of the main text, Eq.(18). The argument is similar to the unfolding in time used by Williams [39], except that networks of spiking neurons are not Markovian. We claim that the above decomposition holds for an arbitrary network architecture including recurrent connections.

Let 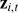 be a collection of discrete random variables, 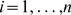 a location index, 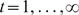 a time index. Denote by 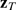 the whole collection up to time 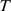. In our example, the index 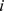 encompasses both the place and action cells. Moreover, 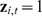 (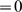) if the corresponding cell did (did not) emit a spike at time 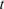. We assume that the sequence is generated by choosing at time 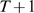 the value 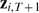 with a probability 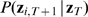. For spiking neurons the sequence 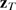 determines the internal states (membrane potentials) at time 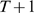 and this modulates the probability of firing at time 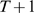 given the previous spike history, 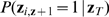. We further assume that the internal stochastic processes which trigger the spikes are independent given the membranes potentials. Hence,
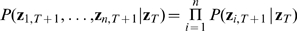
(42)for 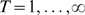.

Because we can always write 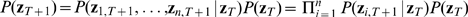 with a factor 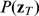, we can iteratively apply an analogous multiplicative decomposition for 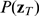, 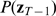, 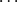, and receive a product representation of 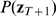. To anchor the product we assume that (42) also holds at 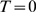, and take this to mean that the initial values 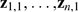 are statistically independent with probabilities given by 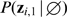. While consecutively applying (42) at each step of the decomposition we arrive at
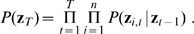
(43)


Setting 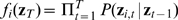 and reordering the product terms we can write (43) as
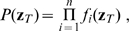
and this is just the decomposition into the product across the place and action cells expressed in (41).

### Implementation

**Figure 9:**
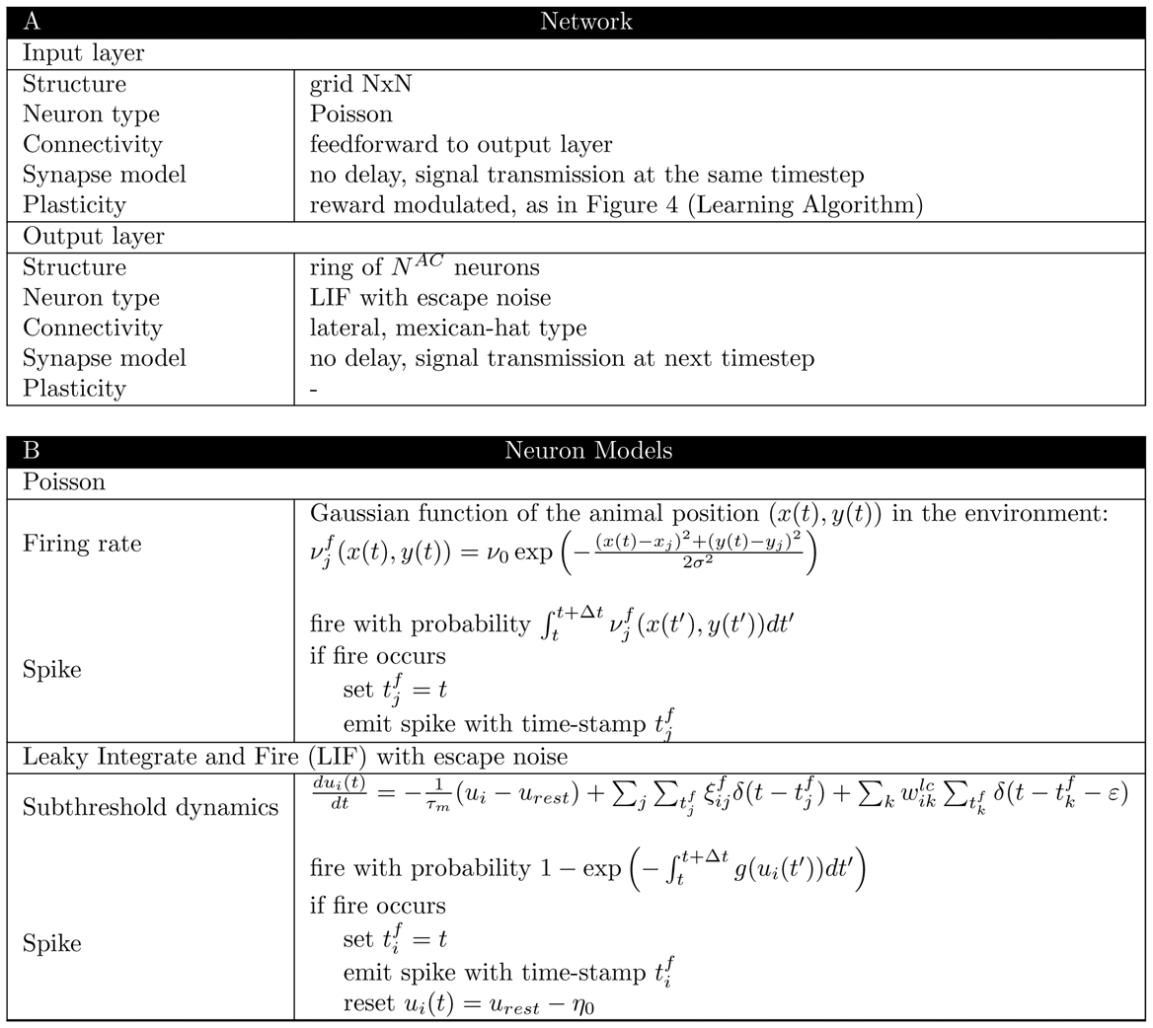
Network description and implementation of neuron models according to [82]. Parameters as in Model architecture, Methods, and Tables 1, 2 (unless otherwise stated in Figure captions).

Model and Figures are produced with Matlab R2008b (Linux version), developed by Mathworks. The model is implemented with custom-made code. For implementation details see Figures 4 and 9. Parameter values are summarized in Tables 1 and 2. The Euler method is used for integration. We discretize the learning rule equation according to the method in paragraph ‘From a single rule to a family of rules’, in order to allow for large time steps. The standard time step in our simulation is 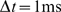. We have checked in additional simulations with smaller time steps of 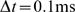 that the results do not depend on the step size (data not shown).

**Table 1:** Parameters for producing the comparison graph of Figure 3.

Panel	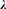	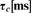	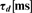	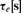	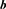	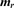	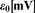	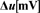
A	0.02	0	200	5	0	-	1	3
B	0.02	0	200	5	0	-	1	5
C	0.02/0.0002	0	200/10	5	0/ 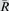	-/150	1/1.3	5
D	0.0002	5	10	5	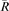	150	1.3	5
E	0.0002	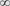	10	5	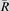	150	1.3	5

Parameter 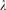 is the learning rate, 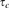 turns the model from a strict policy gradient rule to naive Hebbian, 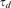 is the time constant used to estimate the firing rate of the action cells, 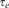 is the time constant of the eligibility trace, 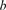 is the reward baseline, 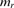 the width of the averaging window of the reward, 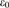 is the height of the postsynaptic pulse produced by the arrival of a spike and 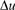 determines the width of the threshold region (escape noise). For C–E integration stops as soon as the total mean firing rate of all action cells 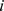, calculated by 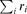, see equation (15), exceeds 200 spikes/ms, i.e. the activity bump is well formed. For panels where two alternative parameter sets are given, both sets give very similar results, and hence we only depict one of them.

**Table 2:** Constant parameters for the Leaky Integrate and Fire neurons.

Model	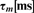	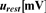	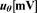	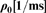	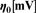
LIF with escape noise (Action Cells)	10	−70	−50	1	5

Parameter 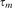 is the membrane time constant, 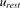 is the resting potential, 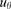 is the formal firing threshold, 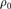 is the stochastic intensity at threshold and 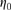 the amount by which the membrane potential is reset after a spike.

## References

[1] Sutton R, Barto A (1998). Reinforcement learning.

[2] Thorndike E (1911). Animal Intelligence.

[3] Rescorla R, Wagner A, Black AH, Prokasy W (1972). A theory of pavlovian conditioning: variations in the effectiveness of reinforecement and nonreinforcement.. Classical Conditioning II: current research and theory.

[4] Klopf A (1982). The hedonistic neuron: a theory of memory, learning, and intelligence.. Hemisphere.

[5] Klopf A (1988). A neuronal model of classical conditioning.. Psychobiology.

[6] Sutton RS, Barto AG (1981). Towards a modern theory of adaptive networks: expectation and prediction.. Psychol Rev.

[7] Barto A, Sutton R, Anderson C (1983). Neuronlike adaptive elements that can solve difficult learning and control problems.. IEEE sys man cybern.

[8] Sutton R, Barto A, Gabriel M, Moore J (1990). Time-derivative models of pavlovian reinforcement.. Learning and Computational Neuroscience: Foundations of Adaptive Networks.

[9] Bliss TVP, Collingridge GL (1993). A synaptic model of memory: long-term potentiation in the hippocampus.. Nature.

[10] Malenka RC, Bear MF (2004). LTP and LTD: An embarassment of riches.. Neuron.

[11] Hebb DO (1949). The Organization of Behavior.

[12] Oja E (1982). A simplified neuron model as a principal component analyzer.. J Math Biol.

[13] Kohonen T (1989). Self-organization and associative memory, 3rd edition.

[14] von der Malsburg C (1973). Self-organization of orientation selective cells in the striate cortex.. Kybernetik.

[15] Bienenstock E, Cooper L, Munroe P (1982). Theory of the development of neuron selectivity: orientation specificity and binocular interaction in visual cortex.. J Neurosci.

[16] Gerstner W, Kempter R, van Hemmen JL, Wagner H (1996). A neuronal learning rule for sub-millisecond temporal coding.. Nature.

[17] Abbott LF, Nelson SB (2000). Synaptic plastictiy - taming the beast.. Nat Neurosci.

[18] van Rossum MCW, Bi GQ, Turrigiano GG (2000). Stable Hebbian learning from spike timing-dependent plasticity.. J Neurosci.

[19] Senn W, Tsodyks M, Markram H (2001). An algorithm for modifying neurotransmitter release probability based on pre- and postsynaptic spike timing.. Neural Computat.

[20] Gerstner W, Kistler WK (2002). Spiking Neuron Models.

[21] Morrison A, Diesmann M, Gerstner W (2008). Phenomenological models of synaptic plasticity based on spike timing.. Biolog Cybern.

[22] Schultz W, Dayan P, Montague R (1997). A neural substrate for prediction and reward.. Science.

[23] Wickens J, Kotter R, Houk J, Davis J, Beiser DG (1995). Cellular models of reinforcement.. Models of information processing in basal ganglia.

[24] Wickens J (1997). Basal ganglia: structure and computations.. Network-Comp Neural.

[25] Reynolds JNJ, Hyland BI, Wickens JR (2001). A cellular mechanism of reward-related learning.. Nature.

[26] Reynolds JNJ, Wickens JR (2002). Dopamine-dependent plasticity of corticostriatal synapses.. Neural Networks.

[27] Frey U, Morris R (1997). Synaptic tagging and long-term potentiation.. Nature.

[28] Reymann KG, Frey JU (2007). The late maintenance of hippocampal LTP: requirements, phases, ‘synaptic tagging’, ‘late-associativity’ and implications.. Neuropharmacology.

[29] Sajikumar S, Frey JU (2004). Resetting of ‘synaptic tags’ is time- and activity-dependent in rat hippocampal ca1 in vitro.. Neuroscience.

[30] Sajikumar S, Navakkode S, Frey JU (2007). Identification of compartment- and process-specific molecules required for ‘synaptic tagging’ during long-term potentiation and long-term depression in hippocampal CA1.. J Neurosci.

[31] Pawlak V, Kerr JND (2008). Dopamine receptor activation is required for corticostriatal spike-timing-dependent plasticity.. J Neurosci.

[32] Xie X, Seung S (2004). Learning in neural networks by reinforcement of irregular spiking.. Phys Rev E.

[33] Florian RV (2007). Reinforcement learning through modulation of spike-timing-dependent synaptic plasticity.. Neural Computat.

[34] Pfister JP, Toyoizumi T, Barber D, Gerstner W (2006). Optimal spike-timing dependent plasticity for precise action potential firing in supervised learning.. Neural Computat.

[35] Izhikevich E (2007). Solving the distal reward problem through linkage of stdp and dopamine signaling.. Cereb Cortex.

[36] Legenstein R, Pecevski D, Maass W (2008). A learning theory for reward-modulated spike-timing-dependent plasticity with application to biofeedback.. PLoS Comput Biol.

[37] Potjans W, Morrison A, Diesmann M (2009). A spiking neural network model of an actor-critic learning agent.. Neural Comput.

[38] Baras D, Meir R (2007). Reinforcement learning, spike-time-dependent plasticity, and the bcm rule.. Neural Comput.

[39] Williams R (1992). Simple statistical gradient-following methods for connectionist reinforcement learning.. Mach Learn.

[40] Baxter J, Bartlett P, Weaver L (2001). Experiments with infinite-horizon, policy- gradient estimation.. J Artif Intell Res.

[41] Farries MA, Fairhall AL (2007). Reinforcement Learning With Modulated Spike Timing Dependent Synaptic Plasticity.. J Neurophysiol.

[42] Kempter R, Gerstner W, van Hemmen JL (1999). Hebbian learning and spiking neurons.. Phys Rev E.

[43] Watkins C (1989). Learning from delayed rewards.

[44] Suri R, Schultz W (2001). Temporal difference model reproduces anticipatory neural activity.. Neural Comput.

[45] Di Castro D, Volkinshtein S, Meir R (2009). Temporal difference based actor critic learning - convergence and neural implementation.. NIPS.

[46] Seung H (2003). Learning in spiking neural networks by reinforcement of stochastic synaptic transmission.. Neuron.

[47] Fiete I, Seung H (2006). Gradient learning in spiking neural networks by dynamic perturbation of conductances.. Phys Rev Lett.

[48] Wörgötter F, Porr B (2005). Temporal sequence learning, prediction, and control: a review of different models and their relation to biological mechanisms.. Neural Comput.

[49] Roberts P (1999). Computational consequences of temporally asymmetric learning rules: I. Differential Hebbian learning.. J Comput Neurosci.

[50] Rao R, Sejnowski T (2000). Predictive sequence learning in recurrent neocortical circuits..

[51] Morris R, Garrard P, Rawlins J, O'Keefe J (1982). Place navigation impaired in rats with hippocampal lesions.. Nature.

[52] Foster D, Morris R, Dayan P (2000). Models of hippocampally dependent navigation using the temporal difference learning rule.. Hippocampus.

[53] Arleo A, Gerstner W (2000). Spatial cognition and neuro-mimetic navigation: a model of hippocampal place cell activity.. Biol Cybern.

[54] Stroesslin T, Sheynikhovich D, Chavarriaga R, Gerstner W (2005). Robust self-localisation and navigation based on hippocampal place cells.. Neural Networks.

[55] Sheynikhovich D, Chavarriaga R, Strösslin T, Gerstner W (2005). Spatial representation and navigation in a bio-inspired robot..

[56] Poucet B, Lenck-Santini PP, Paz-Villagrán VE, Save E (2003). Place cells, neocortex and spatial navigation: a short review.. J Physiology-Paris.

[57] Eichenbaum H, Stewart C, Morris R (1990). Hippocampal representation in place learning.. J Neurosci.

[58] Dayan P (1992). The convergens of TD (*λ*) for general *λ*.. Mach learn.

[59] Dayan P, Sejnowski T (1994). TD(*λ*) converges with probability 1.. Mach Learn.

[60] Tsodyks M, Markram H (1997). The neural code between neocortical pyramidal neurons depends on neurotransmitter release probability.. P Natl Acad Sci USA.

[61] Clopath C, Ziegler L, Vasilaki E, Büsing L, Gerstner W (2008). Tag-trigger-consolidation: a model of early and late long-term-potentiation and depression.. PLoS Comput Biol.

[62] Stein RB (1965). A theoretical analysis of neuronal variability.. Biophys J.

[63] Gerstner W, van Hemmen JL (1992). Associative memory in a network of ‘spiking’ neurons.. Network.

[64] Jolivet R, Rauch A, Lüscher HR, Gerstner W (2006). Predicting spike timing of neocortical pyramidal neurons by simple threshold models.. J Comput Neurosci.

[65] Morris R, Moser EI, Riedel G, Martin SJ, Sandin J (2003). Elements of a neurobiological theory of the hippocampus: the role of activity-dependent synaptic plasticity in memory.. Phil Trans R Soc Lond B.

[66] Morris R (2007). Theories of hippocampal function.. The hippocampus book.

[67] Vasilaki E, Fusi S, Wang XJ, Senn W (2009). Learning flexible sensori-motor mappings in a complex network.. Biol Cybern.

[68] Redgrave P, Gurney K (2006). The short-latency dopamine signal: a role in discovering novel actions?. Nat Rev Neurosci.

[69] Doya K (2002). Metalearning and neuromodulation.. Neural Networks.

[70] Devan B, White N (1999). Parallel information processing in the dorsal striatum: Relation to hippocampal function.. J Neurosci.

[71] Packard M, McGaugh J (1996). Inactivation of hippocampus or caudate nucleus with lidocaine differentially affects expression of place and response learning.. Neurobiol Learn Mem.

[72] White N, McDonald R (2002). Multiple parallel memory systems in the brain of the rat.. Neurobiol Learn and Mem.

[73] Hull C (1943). Principles of behavior.

[74] Toleman E (1948). Cogitiva maps in rats and men.. Psychol Rev.

[75] Wang XJ (2002). Probabilistic decision making by slow reverrberation in cortical circuits.. Neuron.

[76] Zhang JC, Lau PM, Bi GQ (2009). Gain in sensitivity and loss in temporal contrast of stdp by dopaminergic modulation at hippocampal synapses.. Proc Natl Acad Sci USA.

[77] Markram H, Lübke J, Frotscher M, Sakmann B (1997). Regulation of synaptic efficacy by coincidence of postysnaptic AP and EPSP.. Science.

[78] Sjöström PJ, Rancz EA, Roth A, Häusser M (2008). Dendritic excitability and synaptic plasticity.. Physiol Rev.

[79] Loewenstein Y, Seung HS (2006). Operant matching is a generic outcome of synaptic plasticity based on the covariance between reward and neural activity.. Proc Natl Acad Sci USA.

[80] Urbanczik R, Senn W (2009). Reinforcement learning in populations of spiking neurons.. Nat Neurosci.

[81] Barto A (1985). Learning by statistical cooperation of self-interested neuron-like neuron elements.. Hum Neurobiol.

[82] Nordlie E, Gewaltig MO, Plesser HE (2009). Towards reproducible descriptions of neuronal network models.. PLoS Comput Biol.

