# Correction: Spike-Based Reinforcement Learning in Continuous State and Action Space: When Policy Gradient Methods Fail

**DOI:** 10.1371/annotation/307ea250-3792-4ceb-b905-162d86c96baf

**Published:** 2009-12-14

**Authors:** Eleni Vasilaki, Nicolas Frémaux, Robert Urbanczik, Walter Senn, Wulfram Gerstner

In the Funding section, the full name of the funding agency (FACETS) was listed incorrectly. The correct name is: Fast Analog Computing with Emergent Transient States.

